# Individual Socioeconomic Measures and Change in HoNOS Scores among Persons Attending Adult Community Mental Health Services

**DOI:** 10.1007/s10597-026-01595-9

**Published:** 2026-02-11

**Authors:** Vinay Lakra, Anton Isaacs, Tim Powers, Yang Yun, Lokesh Sekharan, Abel Thamby, Veerappa Patil, Srikanth Srikanth, Madushi Senevirathne, Achini Samaranayake, Rachel Mulroy

**Affiliations:** 1 Mental Health, Northern Health, Melbourne, Australia; 2 School of Rural Health, Monash University, Melbourne, Australia; 3 Statistical Consulting Unit, Monash University, Melbourne, Australia; 4 District General Hospital, Ministry of Health, Vavuniya, Sri Lanka

**Keywords:** Mental health services, Social determinants of health, Housing, Employment, Education, caregivers

## Abstract

Socioeconomic disadvantage is associated with poor mental health outcomes. However, whether it also influences treatment outcomes is unclear. The objectives of this study were (1) To measure the impact of mental health services on change in Health of the Nation Outcome Scale [HoNOS] scores between service entry and the last available assessment (median follow-up 24 months; IQR 10–49), (2) To examine if minority or indigenous status influenced change in HoNOS scores and (3) To explore associations between neighbourhood-level and individual-level measures of socioeconomic status [SES] and changes in HoNOS scores. Electronic medical records of 246 adult clients (mean age 36.6 years, 55% male) were analysed from four mental health services of Northern Health in Melbourne, Australia. Exploratory factor analysis (KMO = 0.54; Bartlett’s *p*<.01) identified two individual-level SES measures (individual economic capital and household stability and support). Linear mixed modelling (random intercept) tested associations between individual-level SES and HoNOS, controlling for baseline age, sex, new-consumer status, indigenous status, language and neighbourhood SES indices. Among 246 clients, mean HoNOS scores improved from 11.27 (*SE* = 0.41) at service entry to 9.95 (*SE* = 0.43) at last available follow-up (β = -1.32, *p* < .01). Neither indigenous status nor non-English language predicted change (*p* > .05), and neighbourhood-level SES indices showed no association with HoNOS scores (*p* > .05). In contrast, higher individual economic capital (β = -0.63, *p* < .01) and greater household stability and support (β = -0.85, *p* < .01) were each independently linked to larger improvements in HoNOS scores. These findings indicate that individual-level socioeconomic factors drive improvements in HoNOS scores during engagement with community mental health services.

**Supplementary Information:**

The online version contains supplementary material available at 10.1007/s10597-026-01595-9.

## Background

Indicators of socioeconomic status, such as income and poverty have been shown to be associated with a higher prevalence of mental health problems in developed countries (Allen et al., 2014; Caron & Liu, 2010; Fisher & Baum, 2010; Garratt et al., 2016; Isaacs et al., 2018; Sylvestre et al., 2018). In Australia, people with the lowest income are more likely to experience high levels of psychological distress when compared to those with the highest income (Isaacs et al., 2018). Factors that contribute to this disparity are ‘the conditions in which people are born, grow, live, work, and age’ and are referred to as social determinants (World Health Organization Commission on Social Determinants of Health, 2008). Socioeconomic disadvantage has also been associated with poor access to mental health services (Bartram, 2019; Meadows et al., 2015). It is suggested that interventions to address this disparity need to be carried out upstream, to reduce income inequality and poverty rather than at the clinical and individual level (Compton & Shim, 2015; Kirkbride et al., 2024). However, implementing interventions to reduce income inequality and poverty is challenging.

Treatment goals for persons with mental health challenges have undergone change in the last decade, with a shift in focus from clinical to personal recovery (Commonwealth of Australia, 2013) where the need to meet psychosocial needs of service users in addition to clinical improvements, is gaining traction. Changes such as the introduction of peer workers (Oborn et al., 2019) and more involvement of carers and families in mental health care (Waller et al., 2019) are beginning to materialise. Nonetheless, during the period 2023-24 less than half (49.7%) the number of adult clients who were provided with community mental health care services in Australia showed significant improvement (Australian Government Australian Institute of Health and Welfare, 2024). These reported improvements were clinician rated and were based on measurements using the Health of the Nation Outcome Scales [HoNOS] conducted at enrolment and discharge from the public mental health services (Crawford et al., 2017).

Reasons for low improvement rates could be multiple and evidence suggests that low socio-economic status might play a role (Bennett & Rosenheck, 2021; Elwadhi & Cohen, 2020; Finegan et al., 2018; Mills et al., 2022). While some reports suggest that treatment outcomes in service users are similar, irrespective of their socioeconomic status, (Poots et al., 2014) other studies show that associations do exist between the presence of socioeconomic deprivation and poor outcomes in mental health service users. For instance, education level, (not having a college degree), low income (being unemployed) and belonging to ethnic minority backgrounds have been shown to be associated with poorer responses to treatment (Elwadhi & Cohen, 2020; Mills et al., 2022). Evidence on the relationship between cultural diversity (including being indigenous) and mental health outcomes is still emerging (Jorm et al., 2012).

Furthermore, measurement approaches in monitoring mental health outcomes differ in focus and respondents. While the HoNOS is clinician-rated and assesses consumer symptoms, behaviours and social domains of functioning (Burgess, 2015), consumer-rated measures such as the Kessler-10 and the Mental Health Inventory [MHI-5] measure non-specific psychological distress and well-being (Ten Have et al., 2024). Such broader measures may be more sensitive to environmental stressors and community influences (Kessler et al., 2002).

Cohen (2023) proposes some directions for further research to confirm the association between socioeconomic factors and current treatment outcomes. He suggests that outcomes be considered over the longer term (i.e., 2 years or more) to understand how the effects of social factors might weaken or strengthen over time and that the role of ethnicity as a risk factor be explored further. Consequently, this study explores mental health outcomes (HoNOS scores) measured at two timepoints with aggregated patient data from medical records (Fortney et al., 2017).

### Socio-Economic Status [SES

SES is a multidimensional construct that reflects an individual’s or group’s access to economic resources, social standing, and power. It is typically measured through indicators such as income, education, employment (Diemer et al., 2013; Mayer et al., 2004), cultural capital, and living conditions (Diemer et al., 2013), and captures both material wealth and social standing within a societal hierarchy. SES describes not only the availability of resources and capital to people, but also the likelihood of their exposure to certain risks and harm (Blakemore et al., 2009), such as living in an unsafe environment, lacking access to healthcare, or social isolation. As a determinant of health and opportunity, SES profoundly shapes life outcomes across a person’s lifespan. There is no single best measurement of SES (Williams, 2009), which indicates that researchers need to carefully match their choice of SES measurement to the cohort and phenomenon under investigation (Diemer et al., 2013).

### Measuring SES

#### Income and Employment

Income and employment status has been commonly employed as separate components of SES measurement. While income is often used as a single measure of economic resource (Richardson et al., 2025), there are inherent errors in reporting income. For instance, reported income may overlook government subsidies for temporary or chronic sickness, disability payments, subsidised housing, unemployment subsidies (Diemer et al., 2013), and social support (Thoits, 2011). Higher levels of unemployment and individuals on disability or permanent sickness benefits have demonstrated a substantially strong positive effect with poorer mental health. (Buckman et al., 2022; Fone et al., 2007).

#### Education

Education is also a widely used indicator of SES and a strong indicator of mental health outcomes. Similar to income, education has been used as a single measure of SES (Blakemore et al., 2009), and is often measured by years of education (Diemer et al., 2013). Education has been associated positively with employment, salaries, and ability to afford better housing through the acquisition of skills, information and knowledge (Lynch & Kaplan, 2000). Educational attainment during adolescence has also shown to significantly predict mental health outcomes in adulthood, with lower levels of education associated with higher incidence of depressive symptoms in adults (Jorgensen et al., 2025).

#### Housing

Both the quality of housing and the sharing of accommodation (housing status) have been associated with mental health status. Poor housing quality and hardship in meeting the cost of house maintenance has been related to poorer mental health (Seo & Park, 2021). Housing status has demonstrated that homeowners appear to have better mental health when compared to renters (Park & Seo, 2020), and individuals living with family or friends, residing in hostels, or being homeless (Buckman et al., 2022). However, studies have also shown that co-living can be beneficial to mental health when forming one’s own family (Park & Seo, 2020), or to mitigate isolation, (Santini et al., 2020), and/or reduce stress via emotional support (Thoits, 2011). As such, the context of housing status is important. For instance, where living assistance is needed (e.g., carer assistance) housing status may be related to unemployment and/or greater dependence on social assistance. It is therefore important to capture important dimensions of SES (Galobardes et al., 2006) which better reflect the complexities of socioeconomic status (Blakemore et al., 2009; Fone et al., 2007).

#### Levels of Measurement

Socioeconomic status can be measured at the individual level, at the household level, or for the neighbourhood of residence (Cheng et al., 2024; Pickett & Pearl, 2001). Neighbourhood SES is widely used in population health research not only because neighbourhood contexts may exert independent effects on health, but also for pragmatic reasons, as area-level measures provide a readily available proxy for multiple individual-level socioeconomic characteristics when such data are unavailable or incomplete (Diez Roux, 2001; Galobardes et al., 2006). An important and unresolved empirical question is the extent to which neighbourhood SES functions as a valid surrogate for individual-level SES, versus capturing distinct contextual influences—an issue directly addressed by the present study.

Neighbourhood measures of SES are often developed using Census data and can be useful in capturing the more stable influence of the socioeconomic environment a resident experiences in their daily lives. Such information can inform public health policy and funding distribution (Krieger et al., 1997). While useful at a global level, such measures can be misleading when interpreted at the individual level (Blakemore et al., 2009).

Choosing SES measures can also be problematic when trying to disentangle neighbourhood effects from influences specific to the individual. For instance, some researchers (Fone et al., 2007; Pickett & Pearl, 2001), have found that individual measures of SES demonstrated substantially stronger effects on individual mental health when compared to neighbourhood measures of SES. Such underestimations of neighbourhood SES may be attributable to situations where there is substantial socioeconomic heterogeneity in the neighbourhood (Nguyen et al., 2023). Community mental health services routinely collect consumer data on socioeconomic factors such as cultural and linguistic background, housing, employment, education, and benefit status.

This study aimed to measure clinical outcomes over time among persons aged 18 years and over who were registered with Community Mental Health Services and their association with socio-economic factors. Specific objectives of the study were as follows:

1. To measure the impact of mental health services on change in HoNOS scores between service entry and the last available assessment (median follow-up 24 months).
2. To examine if minority or indigenous status influenced change in HoNOS scores.
3. To explore associations between neighbourhood-level and individual-level measures of SES and changes in HoNOS scores.

## Methodology

### Setting

Northern Health is the major provider of public health care in Melbourne’s rapidly growing outer north, covering the local government areas of Moreland, Darebin, Whittlesea and Hume (Northern Health, 2024b). Its mental health division operates adult community mental health services at South Preston, Broadmeadows, Coburg and Mill Park (Northern Health, 2024a). When compared to Greater Melbourne, these geographical areas experience greater disadvantage characterised by higher rates of unemployment and a larger proportion of low-income households.

### Participants

De-identified data were extracted from Electronic Medical Records and archived case records of clients from the four community mental health services of Northern Health. The sampling frame comprised all adult clients (aged 18–64 years) registered with Northern Health adult community mental health services from 1 st January 2021–31st December 2023 who had HoNOS assessments for at least two time points (at service entry and the last available).

An a priori sample size calculation was conducted in G*Power 3.1 (Faul et al., 2009), for linear multiple regression to ensure 80% power (1 – β = 0.80) at α = 0.05 for detecting a medium-small effect [f² = 0.07; (Cohen, 1988)] with up to 20 predictors. This sample size was then adjusted for the repeated measures in the linear mixed modelling using the Design Effect (Deff; Snijders, 2005) with an estimated intraclass correlation of 0.25. The final sample size required was 198 participants, which was increased to a target sample size of 264 to allow for missing values. Although, this sample size is not adequate to detect small effect sizes (i.e., f² = 0.02 would require a sample of 665 clients) the literature suggests that the relationship between health outcomes and SES is medium to strong, which implies that a larger sample size was not required.

An electronic medical record query identified *n* = 66 potentially eligible clients from each of the four community teams. Because several socioeconomic indicators required manual extraction and harmonisation from routine EMR fields, a priori target sampling was used to define a feasible chart-review workload while minimising selection bias (Hoenig & Heisey, 2001). We therefore drew a simple random sample stratified by clinic (proportional allocation).

Clients were randomly selected from community teams proportionally to represent the composition of Northern Health Community Mental Health Services. Of the *n* = 264 sampled records, 15 were excluded because HoNOS was not available at both enrolment and last available HoNOS score (i.e., 2 timepoints), and 3 were excluded due to clear data-entry errors in HoNOS values. The final analytic sample comprised *n* = 246 consumers. The mean age of clients was 36.6 years (SD = 11.8) when they first entered the study. Just over half were male (54%).

### Measures

Demographic and socioeconomic data from Northern Health community mental health services that were routinely collected during the study period, were collated together with HoNOS scores at service entry and last available assessment.

#### Mental Health

Mental health was measured using the HoNOS (Wing et al., 1998). HoNOS is a clinician-rated outcome measure for working-age adults (18–64 years) in contact with mental health services. It is a widely used, validated measure of mental health and functioning (Crawford et al., 2017), that is nationally mandated for use by Australian public mental health services at admission, review and discharge to measure mental health and social functioning of individual clients. It comprises 12 items covering behaviour, impairment, symptoms and social functioning; each item is scored from 0 (no problem) to 4 (severe to very severe problem) based on the worst severity over the preceding two weeks. Total scores on the HoNOS scale can range between 0 and 48. These scores are used to monitor change over time and evaluate service effectiveness. When the magnitude of change measured by the effect size statistic, is greater than or equal to + 0.5, it is considered to be a significant improvement in mental health and social functioning (Burgess et al., 2015). HoNOS scores from two time points for each participant were included in this study – the initial score recorded at service entry and the latest available score (median follow-up 24 months; IQR 10–49). Data from the consumer-reported Behaviour and Symptom Identification Scale [BASIS 32] was not collected as completion rates were very low.

#### Background and Characteristics

*Age* was measured using the consumer’s reported date of birth. *Sex* was self-reported by clients. Male (*n* = 133) and female (*n* = 110) the predominant identity, with 3 identifying as trans or non-binary. *New clients* were identified at the baseline measure to control for differences over time which may be confounded by those with prior experience at the clinic. Minority status was identified by two separate binary measures: (1) *indigenous* clients (i.e., Aboriginal or Torres Strait Islander), and (2) clients who spoke a *language other than English* at home.

#### SES

*Neighbourhood SES* was quantified using three indices derived from the Australian Bureau of Statistics 2021 Census data: Index of Relative Socio-economic Advantage and Disadvantage, Index of Economic Resources and Index of Education and Occupation (Australian Bureau of Statistics, 2023). These were mapped to each consumer’s residential postcode. Each index is standardized to a mean of 1000 (*SD* = 100), with higher values indicating greater neighbourhood advantage, resource availability or educational/occupational attainment. However, neighbourhood-level SES indices were rescaled by dividing by 100 to aid interpretability of regression coefficients (i.e., coefficients represent the expected change in outcome per 100-unit increase in the original SES index). These continuous scores serve as area-level SES covariates, distinct from the individual-level SES proxies described below.

*Individual-level SES* was measured by creating a composite score(s) from six SES proxy measures. These measures included *benefit payments* (e.g., disability, sickness), *living status* (e.g., living alone, with siblings), *housing arrangement* (e.g., house, boarding), *carer need* (e.g., carer not needed, lives alone with a carer), *employment status* (e.g., employed, student), and highest level of attained *education* (e.g., secondary, vocational, tertiary). Since these indicators were recorded as multi-category fields with heterogeneous response options, we harmonised them to a common 5-level ordered scale (1 = *very low independence* to 5 = *very high independence*) intended to reflect relative socioeconomic advantage/independence. See Supplementary file 1. This recoding was theory-informed (i.e., consistent with conventional socioeconomic gradients) and designed to permit construction of a parsimonious composite SES measure using principal component analysis (PCA); PCA based SES indices using discrete proxy indicators are widely used, with known assumptions and limitations (Kolenikov & Angeles, 2009).

Constructing a composite socioeconomic proxy by harmonising categorical indicators and combining them using dimension-reduction methods is common in global health and development economics (Filmer & Pritchett, 2001; Vyas & Kumaranayake, 2006), epidemiology and public health (Galobardes et al., 2006), and health services research using routinely collected clinical/administrative datasets where individual SES is not routinely captured (Johnson et al., 2013; Takahashi et al., 2016). We acknowledge that any manual harmonisation imposes assumptions about ordering and spacing of categories; therefore, we interpret the resulting score as a pragmatic SES proxy.

### Analytical Plan

An exploratory factor analysis was conducted on the six proxy measures surmised to be related to individual-level SES to ascertain whether or not individual SES was a single component (i.e., using principal component analysis). Sampling adequacy was determined by assessing the Kaiser-Meyer-Olkin measure [KMO]. KMO between 0.5 and 0.7 is considered adequate (Beavers et al., 2013; Hair et al., 2014) and a significant Bartlett Test of Sphericity indicates sufficient inter-item correlations exist to justify factor analysis (Beavers et al., 2013). The number of components was assessed by ensuring that indicators primarily loaded on one component, and by inspecting the solutions with one or more components for eigenvalues greater than 1 (Zwick & Velicer, 1986). As a sensitivity check to assess the robustness of the SES construct to alternative measurement assumptions, we also evaluated an ordinal factor model based on polychoric correlations, which has been used when indicators are ordinal. This analysis was undertaken to examine whether the substantive SES structure was sensitive to assumptions about category spacing, rather than as a replacement for the primary PCA-based composite used in subsequent analyses, which was retained to support parsimonious modelling and comparability with prior health services research.

The research questions were answered using linear mixed modelling (LMM) to control for repeated HoNOS measurements along with a range of predictors (both within and between predictors). Hierarchical LMM was used to answer each research question in a stepped manner. That is, the first step explored whether mental health (measured by HoNOS scores) for clients differed over time when controlling for baseline age, sex, and new consumer status. The second step additionally, explored whether identifying as indigenous or speaking a language other than English at home was associated with lower HoNOS scores. And finally, controlling for these prior factors, the neighbourhood- and individual-level of SES (measured at each timepoint) were explored for their relationship to mental health scores across follow-up. Assumptions for normality were conducted by inspecting a histogram of residuals, while homoscedasticity was ascertained by inspecting a scatterplot of the residuals x predicted values (Tabachnick & Fidell, 2019), acknowledging that larger sample sizes are robust against non-normality and heteroscedasticity (Altman & Bland, 1995; Elliott & Woodward, 2007). SPSS Statistics version 29 (IBM Corp., 2022), was used for all analysis. Missingness for routinely collected demographic and SES indicators was low (i.e., < 5%). Given the primary outcome requirement (HoNOS at both timepoints) and the small number of exclusions due to missing HoNOS, no imputation of HoNOS outcomes was undertaken (Sterne et al., 2009). Approval for this study was obtained from the Research Development and Governance Unit of Northern Health (ALR No: 34.2023; Dated 24/7/2023).

## Results

The exploratory factor analysis utilising the proxy measures for individual-level SES indicated sample adequacy (KMO = 0.54) and suitable variance (Bartlett’s Test, *p*<.001). Two components extracted eigenvalues greater than 1, and produced a simple structure for the rotated component matrix where each indicator primarily loaded on one component (see Table 1).

The resulting two components incorporated three indicators each and were interpretable (see Supplementary file 2 for scoring template). The components were named:

- *Individual Economic Capital*: Benefit payment, Employment status, Education level, and
- *Household Stability and Support*: Living status, Housing arrangement, Carer needs.

**Table 1:** Rotated component matrix^a^

Proxy measures	Component	
	1	2
Benefit payment	0.75	
Living status		0.66
Housing arrangement		0.78
Carer need		0.55
Employment status	0.61	
Education level	0.64	

Component loadings < 0.3 are constrained

^a^ Principal Component Analysis conducted with a Quartimax rotation

The sensitivity analysis using ordinal factor analysis yielded a two-factor solution with a substantively consistent loading pattern to the primary PCA, distinguishing economic indicators (benefit receipt, employment, education) from housing and living-context indicators. Because these dimensions were correlated and the study focus was on a general socioeconomic gradient, the PCA-derived composite was retained for the main analyses.

The descriptive statistics are presented in Table 2. Almost half of the clients were new to the clinics at the first timepoint. Indigenous or Torres Strait clients represented 12% of the sample, and 20% spoke a language other than English at home. Neighbourhood SES levels were similar to the Australian population (i.e., population median values = 1000). Although the individual-level SES measures did not have normative scores, it does appear that *household stability and support* was high (*M* = 3.92) on the scale of 1 to 5. *Individual economic capital* appeared to be lower (*M* = 2.16) on a similar scale.

**Table 2:** Spearman correlations and descriptive statistics

Item	1	2	3	4	5	6	7	8	9	10	11
1. Mental Health (HoNOS)											
2. Age	−0.05										
3. Sex (1=male)	0.02	0.02									
4. New client	−0.03	0.04	0.00								
5. Indigenous	0.01	0.06	0.09*	0.05							
6. Non-English home language	−0.04	0.11*	−0.09*	0.06	0.02						
7. Adv/Disadv score (neighbourhood) ^a^	0.03	−0.07	0.03	0.09	−0.08	−0.19*					
8. Resource score (neighbourhood) ^a^	−0.03	−0.01	0.00	−0.10*	−23*	0.01	0.18*				
9. Educ Occ score (neighbourhood) ^a^	0.05	−0.05	0.03	−0.11*	−0.15*	−0.18*	0.95*	0.08			
10. Individual Economic Capital	−0.09*	−0.18*	−0.17*	−0.12*	−0.12*	0.08	−0.06	0.16*	−0.12*		
11. Household stability and support	−0.12*	0.12*	−0.19*	0.06	−0.03	0.08	0.03	−0.02	0.02	0.03	
*M*	10.67	36.61	0.55	0.46	0.12	0.20	0.99	0.98	1.01	2.16	3.92
*SD*	6.45	11.79	0.50	0.50	0.33	0.40	0.72	0.46	0.71	1.25	0.79

*M = Mean; SD = Standard Deviation*

^*a*^ *Neighbourhood SES predictors were divided by 100 prior to modelling*

Linear mixed modelling was conducted (i.e., with random intercept) to inspect the relationship between mental health levels (measured by HoNOS score) with background variables, minority proxy measures, and neighbourhood- and individual-level measures of SES. This was done in using hierarchical regression in four steps (see Table 3). The residuals for all models were inspected (i.e., histograms and scatterplots x predicted results) and found to be normally distributed around zero, and relative homoscedastic (See Supplementary file 3). The intraclass correlation (ICC) for the repeated measure of HoNOS was 0.38 supporting the need to account for repeated measures.

**Table 3:** Fixed effect coefficients predicting mental health (*N* = 246)

	Step 1	Step 2	Step 3	Step 4
	β	β	β	β
Step 1: Background variables^a^				
Time	−1.32*	−1.26*	−1.24*	−1.23
Age	−0.03	−0.03	−0.03	−0.04
Sex (1 = Male)	0.31	0.42	0.41	0.05
New client	−0.77	−0.78	−0.81	−0.92
Step 2: Minority				
Indigenous		−0.41	−0.44	−0.30
Home language		−0.25	−0.24	−0.09
Step 3: Neighbourhood-level SES				
Advantage-Disadvantage score ^b^			−0.06	−0.54
Resource score ^b^			−0.4401	−0.19
Education Occupation score ^b^			0.49	1.20
Step 4: Individual-level SES				
Individual Economic Capital				− 0.63*
Household Stability & Support				− 0.85*

^a^ All models controlled for the four locations using fixed effects. These were excluded to simplify presentation

^b^ Neighbourhood SES predictors were divided by 100 prior to modelling; coefficients therefore represent the association per 100-unit increase in the original SES index

* *p* <.01

In Step 1, there was a statistically significant improvement in average mental health score over the two time points (β = −1.32). On average, consumer HoNOS scores reduced from 11.27 (*SE* = 0.41) at service entry to 9.95 (*SE*=0.43) the last available measure. There was no detected relationship between mental health with gender, age, and whether a consumer was new to the practice at the first timepoint. In Step 2, after adjusting for these covariates, neither Indigenous status nor non-English language spoken at home predicted mental health outcome scores. Step 3 indicated no significant relationship between neighbourhood-level SES measures and mental health scores.

Finally, in Step 4, both individual-level SES indicators—*individual economic capital* and *household stability and support*—were significantly associated with mental health outcomes: greater economic capital and household stability/support corresponded with improved mental health outcomes, independent of neighbourhood SES and background characteristics. Neighbourhood- and individual-levels of SES proved to be dynamic. As such, several clients experienced different levels of SES across the observation period. Hence, the relationship between individual-level SES and mental health was not related to time.

## Discussion

This study explored the change in clinician-rated HoNOS scores between service entry and the last available assessment among 246 adult clients, registered with community mental health services and the association of this change with measures of SES. The results indicated a modest but statistically significant improvement in clinician-rated mental health (HoNOS score) over the period between service entry and the last follow-up, indicating that service engagement yielded meaningful improvement in mental health and functioning. This benefit was equivalent for Indigenous and non-Indigenous clients, as well as for those speaking a language other than English at home, suggesting that the services were culturally and linguistically effective across these subgroups. This finding concurs with those of a previous study mentioned earlier (Poots et al., 2014).

However, contrary to expectations from environmental and social models of health, postcode-based neighbourhood SES indices (Advantage–Disadvantage, Economic Resources, Education–Occupation) did not predict HoNOS trajectories. This finding implies that neighbourhood-level proxies at the postcode scale may lack the granularity required to capture the lived socioeconomic conditions that directly affect mental health (Nguyen et al., 2023). In contrast, both individual-level SES components—*Economic Capital* (*β* = − 0.63, *p* <.01) and *Household Stability & Support* (*β* = − 0.85, *p* <.01) were robustly associated with improved mental health, highlighting the primacy of personal material resources and domestic support networks in shaping clinical outcomes (Fone et al., 2007; Galobardes et al., 2006).

There may be a range of mechanisms that explain the differential effects for individual-level proxy measures of SES. *Individual Economic Capital* may reduce financial stress and enable greater access to psychosocial resources. These may include transport to clinics, affordability of medication, and the ability to access and understand health related information (provided by clinicians or publicly sourced).

*Household stability and support* may operate by reducing stress through stable living arrangements, and social support. Reliable care giving may mitigate isolation and foster adherence to medication regimes (Santini et al., 2020), while emotional support via housing and living arrangements may enhance individual self-efficacy and reductions in stress due to social interactions that provide empathetic listening (Thoits, 2011).

Neighbourhood measures of SES appear unable to distinguish between households with different levels of financial and educational resources levels within the same area (Nguyen et al., 2023; Pickett & Pearl, 2001). One explanation may lie with the measure of mental health utilised within clinics. HoNOS, as a clinician-rated measure of recent symptoms and social functioning directly reflects personal material and psychosocial resources and may be less sensitive to broader contextual features captured in neighbourhood measures of SES (Burgess et al., 2015). In contrast, self-reported scales such as Kessler-10 (Kessler et al., 2002), which measures non-specific psychological distress, and the Mental Health Inventory [MHI-5] (Ten Have et al., 2024), which assesses overall well-being and distress, may be more sensitive to broader environmental influences including neighbourhood poverty and social interaction (Fone et al., 2007). Taken together, these results underscore the importance of assessing material and psychosocial resources at the individual level when evaluating mental health interventions. They also caution against over-reliance on broad area-based SES measures, which may obscure critical heterogeneity in clients’ lived experiences.

These findings indicate that individual-level socioeconomic factors, rather than neighbourhood-based proxies, drive improvements in clinician-rated mental health outcomes over two years of engagement with community mental health services, thereby supporting the case for routine assessment of personal SES factors to inform tailored interventions. The evidence for interventions to address SES needs of mental health service users is still emerging (Baldwin et al., 2025; Isaacs et al., 2019). Care coordination (service coordination) has shown to be useful when multiple agencies have to work together to address these needs (Isaacs et al., 2019; Isaacs & Duncan, 2025).

### Limitations

Some suggest that data from HoNOS may need to be interpreted cautiously. Random variation and subtle changes in practice and case-mix may have led to changes in clients’ outcomes over time. Although our analysis controlled for differences between the four clinics, separating real and spurious differences can be difficult (Powell et al., 2003). Furthermore, we acknowledge that individual SES proxies,– while more predictive than postcode derived SES, are themselves simplifications of complex socioeconomic realities.

## Conclusions

These findings indicate that individual-level socioeconomic factors, rather than neighbourhood-based proxies, drive improvements in HoNOS scores during engagement with community mental health services, thereby supporting the case for routine assessment of personal SES factors to inform tailored interventions.

## Supplementary Information

Below is the link to the electronic supplementary material.

Supplementary Material 1

Supplementary Material 2

Supplementary Material 3

## Data Availability

The datasets generated and/or analysed during the current study are not publicly available since they were obtained from Electronic Medical Records that belong to Northern Health Division of Mental Health.
