# Supplementary material for: Individual Socioeconomic Measures and Change in HoNOS Scores among Persons Attending Adult Community Mental Health Services
Source: Community Ment Health J. 2026 Feb 11;62(6):1084–94. doi: 10.1007/s10597-026-01595-9 (PMC13451511; doi:10.1007/s10597-026-01595-9)
Supplement: Supplementary file 1 [file 10597_2026_1595_MOESM1_ESM.docx]

**Supplementary file 1**

**Article title**: Individual socioeconomic measures and change in HoNOS scores among persons attending adult community mental health services

**Journal name**: Social Psychiatry and Psychiatric Epidemiology

**Author names**: Vinay Lakra, Anton Isaacs, Tim Powers, Yang Yin, Lokesh Sekharan, Abel Thamby, Veerappa Patil, Srikanth Srikanth, Madushi Senevirathne, Achini Samaranayake, Rachel Mulroy

**Corresponding author**: Anton Isaacs, Monash University School of Rural Health, Warragul, Victoria, Australia. Email: anton.isaacs@monash.edu

**Coding of Individual-level SES Items**

The following table lists the selection options and coding for questions/items utilised in the individual-level SES measure. The scaling for all measures ranges from 1-Very Low to 5-Very High.

**Table A1**

*Explanation of Scaling for SES Items*

| **Variable** | **Scaling** | **Reasoning** |
| --- | --- | --- |
| **Benefit payment** |  |  |
| *Disability* | 1-Very Low Independence | Ongoing support needs - minimal independence |
| *Other pension/benefit* | 2-Low Independence | Long‑term financial support - reduced autonomy |
| *Sickness benefit* | 3-Moderate Independence | Temporary health support - some dependency |
| *Unemployment benefits* | 4-High Independence | Transitional support - still largely independent |
| *None* | 5-Very High Independence | No benefits - maximum self‑sufficiency |
| **Living Status** |  |  |
| *Others in care arrangement* | 1-Very Low Independence | Formal care setting - high dependency |
| *Residential full support* | 1-Very Low Independence | Institutional care with 24/7 support |
| *Residential limited support* | 1-Very Low Independence | Structured care environment - low autonomy |
| *Residential no support* | 1-Very Low Independence | Institutional residence with no self‑care responsibility |
| *Living with parents* | 2-Low Independence | Adult reliant on parental household |
| *Parents/Defacto/husband/wife and children* | 2-Low Independence | Multigenerational care/responsibility |
| *Children dependent* | 2-Low Independence | Dependent children require significant support |
| *Living with siblings* | 3-Moderate Independence | Shared family setting - some mutual support |
| *Defacto/husband/wife and children* | 3-Moderate Independence | Caring for dependents introduces additional resources |
| *Other relatives* | 3-Moderate Independence | Extended family household - mixed support/responsibility |
| *Other* | 3-Moderate Independence | Unspecified default to middle category |
| *Defacto/husband/wife* | 4-High Independence | Standard adult partnership; shared resources but self‑sufficient |
| *Children nondependent* | 4-High Independence | Dependent presence does not reduce adult’s autonomy |
| *Lives alone* | 5-Very High Independence | Fully autonomous with no cohabitants |
| *Living with friends* | 5-Very High Independence | Voluntary, peer‑based living - minimal dependency |
| **Housing arrangement** |  |  |
| *Residential care services* | 1-Very Low Independence | Institutional care facility - high dependency |
| *No usual residence* | 1-Very Low Independence | Homelessness/extreme housing instability |
| *Supported residential service* | 2-Low Independence | Regular support provided |
| *Community residential service* | 2-Low Independence | Group living with scheduled assistance |
| *Hostel type accommodation* | 3-Moderate Independence | Shared/transitional housing with oversight |
| *Other accommodation* | 4-High Independence | Varies, but generally non‑institutional |
| *Boarding* | 4-High Independence | Semi‑independent lodging; some reliance on host |
| *House/flat* | 5-Very High Independence | Private dwelling under occupant control |
| *Caravan* | 5-Very High Independence | Mobile but self‑managed housing |
| **Carer availability** |  |  |
| *Lives with another has a residential carer* | 1-Very Low Independence | Dual dependency: household and formal care provision |
| *Lives alone has a carer* | 2-Low Independence | Dependent on personal carer despite solitary residence |
| *Boarding* | 3-Moderate Independence | Partial reliance on host for daily needs |
| *Lives with another has no carer* | 4-High Independence | Mutual informal support; formal carer not required |
| *Carer not needed* | 5-Very High Independence | Fully self‑sufficient; no support needed |
| *Lives alone has no carer* | 5-Very High Independence | Independence maintained despite solitary living |
| **Employment** |  |  |
| *Unemployed* | 1-Very Low Independence | No income; reliance on welfare |
| *Pensioner* | 1-Very Low Independence | Government pension indicates high dependency |
| *Retired* | 2-Low Independence | Dependence on pensions/savings |
| *Home duties* | 3-Moderate Independence | Reliance on partner’s/household income |
| *Student* | 4-High Independence | Partial autonomy; some reliance on support |
| *Employed* | 5-Very High Independence | Earned income; economic self‑sufficiency |
| **Education** |  |  |
| *KG and Primary* | 1-Low educational attainment | Basic schooling only |
| *Secondary 7-10* | 1- Low educational attainment | Incomplete secondary education |
| *Secondary 11-12* | 2-Basic secondary attainment | Completed high school |
| *Vocational* | 3-Intermediate vocational skills | Trade/technical training |
| *Tertiary commenced* | 4-Adv post-secondary studies | Some tertiary attainment |
| *Tertiary completed* | 5-Highest tertiary qualification | Highest formal qualification |
