# Supplementary material for: Individual Socioeconomic Measures and Change in HoNOS Scores among Persons Attending Adult Community Mental Health Services
Source: Community Ment Health J. 2026 Feb 11;62(6):1084–94. doi: 10.1007/s10597-026-01595-9 (PMC13451511; doi:10.1007/s10597-026-01595-9)
Supplement: Supplementary file 2 [file 10597_2026_1595_MOESM2_ESM.docx]

**Supplementary file 2**

**Article title**: Individual socioeconomic measures and change in HoNOS scores among persons attending adult community mental health services

**Journal name**: Social Psychiatry and Psychiatric Epidemiology

**Author names**: Vinay Lakra, Anton Isaacs, Tim Powers, Yang Yin, Lokesh Sekharan, Abel Thamby, Veerappa Patil, Srikanth Srikanth, Madushi Senevirathne, Achini Samaranayake, Rachel Mulroy

**Corresponding author**: Anton Isaacs, Monash University School of Rural Health, Warragul, Victoria, Australia. Email: anton.isaacs@monash.edu

**Individual-level SES Components**

The following table lists the selection options and scoring for the two components of individual-level SES measure: (1) *Individual Economic Capital*, and (2) *Household Stability and Support*. The scaling for all measures ranges from 1-Very Low to 5-Very High. The logic for each level of scaling is provided for each response. Measure of Individual Economic Capital includes Benefit payment, Employment and Education. Measure of Household Stability and Support includes Living status, Housing arrangement and Carer availability.

**Table B1**

*Scoring for Individual-level SES Components*

| **Variable** | **Scaling** | ***Individual Economic Capital*** | ***Household Stability and Support*** |
| --- | --- | --- | --- |
| **Benefit payment** (enter score for selection) |  |  |  |
| *Disability* | 1 | √ |  |
| *Other pension/benefit* | 2 | √ |  |
| *Sickness benefit* | 3 | √ |  |
| *Unemployment benefits* | 4 | √ |  |
| *None* | 5 | √ |  |
| **Employment** (enter score for selection) |  |  |  |
| *Unemployed* | 1 | √ |  |
| *Pensioner* | 1 | √ |  |
| *Retired* | 2 | √ |  |
| *Home duties* | 3 | √ |  |
| *Student* | 4 | √ |  |
| *Employed* | 5 | √ |  |
| **Education** (enter score for selection) |  |  |  |
| KG and Primary | 1 | √ |  |
| Secondary 7-10 | 1 | √ |  |
| Secondary 11-12 | 2 | √ |  |
| Vocational | 3 | √ |  |
| Tertiary commenced | 4 | √ |  |
| Tertiary completed | 5 | √ |  |
| **Living Status** (enter score for selection) |  |  |  |
| *Others in care arrangement* | 1 |  | √ |
| *Residential full support* | 1 |  | √ |
| *Residential limited support* | 1 |  | √ |
| *Residential no support* | 1 |  | √ |
| *Living with parents* | 2 |  | √ |
| *Parents/Defacto/husband/wife and children* | 2 |  | √ |
| *Children dependent* | 2 |  | √ |
| *Living with siblings* | 3 |  | √ |
| *Defacto/husband/wife and children* | 3 |  | √ |
| *Other relatives* | 3 |  | √ |
| *Other* | 3 |  | √ |
| *Defacto/husband/wife* | 4 |  | √ |
| *Children nondependent* | 4 |  | √ |
| *Lives alone* | 5 |  | √ |
| *Living with friends* | 5 |  | √ |
| **Housing arrangement** (enter score) |  |  |  |
| *Residential care services* | 1 |  | √ |
| *No usual residence* | 1 |  | √ |
| *Supported residential service* | 2 |  | √ |
| *Community residential service* | 2 |  | √ |
| *Hostel type accommodation* | 3 |  | √ |
| *Other accommodation* | 4 |  | √ |
| *Boarding* | 4 |  | √ |
| *House/flat* | 5 |  | √ |
| *Caravan* | 5 |  | √ |
| ***Carer*** ***availability*** (enter score for selection) |  |  |  |
| *Lives with another has a residential carer* | 1 |  | √ |
| *Lives alone has a carer* | 2 |  | √ |
| *Boarding* | 3 |  | √ |
| *Lives with another has no carer* | 4 |  | √ |
| *Carer not needed* | 5 |  | √ |
| *Lives alone has no carer* | 5 |  | √ |
| ***AVERAGE*** |  |  |  |
|  |  | *Individual Economic Capital* | *Household Stability and Support* |
