# Supplementary material for: Individual Socioeconomic Measures and Change in HoNOS Scores among Persons Attending Adult Community Mental Health Services
Source: Community Ment Health J. 2026 Feb 11;62(6):1084–94. doi: 10.1007/s10597-026-01595-9 (PMC13451511; doi:10.1007/s10597-026-01595-9)
Supplement: Supplementary file 3 [file 10597_2026_1595_MOESM3_ESM.docx]

**Supplementary file 3.**

Model diagnostic plots for linear mixed models

This appendix presents graphical diagnostic plots used to assess key assumptions of the linear mixed models reported in the main analyses. Consistent with standard practice, model residuals were inspected to evaluate approximate normality and homoscedasticity, rather than relying on formal statistical tests, which can be overly sensitive in large samples. The plots indicate no substantial departures from assumptions likely to materially affect inference.

**Figure S1.** Histogram and Q–Q plot of conditional residuals from the linear mixed model.

**Figure S2.** Residuals versus fitted values from the linear mixed model, used to assess homoscedasticity and systematic patterns.
